# Gene- and Isoform-Level Responses to Extreme Acidic pH Stress in an Emerging Marine Invertebrate Model Organism *Litoditis marina*

**DOI:** 10.3390/antiox15070862

**Published:** 2026-07-09

**Authors:** Beining Xue, Pengchi Zhang, Hanwen Yang, Liusuo Zhang

**Affiliations:** 1 Laboratory of Experimental Marine Biology, Institute of Oceanology, Chinese Academy of Sciences, Qingdao 266071, China; 2 Laboratory for Marine Biology and Biotechnology, Qingdao Marine Science and Technology Center, Qingdao 266237, China; 3 Center for Ocean Mega-Science, Chinese Academy of Sciences, 7 Nanhai Road, Qingdao 266071, China; 4 University of Chinese Academy of Sciences, Beijing 100049, China; 5 Department of Biological Science and Technology, Jinzhong University, 199 Wenhua Road, Jinzhong 030619, China

**Keywords:** marine nematode, oxidative stress, mitochondria, ferroptosis

## Abstract

Ocean acidification poses a critical threat to marine invertebrate survival and diversification, yet the post-transcriptional regulatory mechanisms underlying acid stress responses remain poorly understood. Here, we employed Oxford Nanopore long-read RNA sequencing to systematically characterize transcriptional and post-transcriptional responses to acidic pH stresses in the marine nematode *Litoditis marina*. Our analysis revealed 912 upregulated and 728 downregulated genes enriched in autophagy, fatty acid metabolism, and peroxisome activation, alongside 327 differential alternative splicing events and 1512 transcripts with significant usage changes under severe acidic pH stress. By integrating weighted gene co-expression network analysis with databases such as WormExp2, we found that acidic pH stress response exhibited resemblance to oxidative stress response. Specifically, we identified genes involved in the oxidative stress response, such as gpx-1, cyp-13A11, trxr-1, cyc-1, and key regulators, including *hlh-30*/TFEB. Genes such as *gpx-1*, *trxr-1,* and *cyp-23A1* might protect *L. marina* from oxidative stress under acidic pH. Moreover, several ferroptosis-related genes, such as *gpx-5*, *fat-2*, and *smf-1*, might render *L. marina* vulnerable to acidic pH stress. Among the genes with splicing changes, we identified oxidative stress responding genes such as *sod-4*, *prx-2*, *coq-2*, *coq-3*, *prx-10*, *ctl-2*, *gst-7*, *trx-1*, *mdt-15*, and *fat-2*. Additionally, we discovered the preference for proximal 3′ UTR under acidic pH stress. Genes related to ferroptosis, including *cyp-23A1*, *C07E3.9*/PLA2G1B, and *C53D5.5*/GGT1, exhibited differential 3′ UTR usage under acidic pH stress. Our findings shift the focus from traditional gene-centric analyses to capturing the full breadth of post-transcriptional diversity, providing novel insights into post-transcriptional gene regulation in marine metazoans under environmental stress, as well as revealing that alleviating oxidative stress might increase resistance to acid pH stress.

## 1. Introduction

Increased atmospheric CO_2_-induced ocean acidification (OA) threatens the marine calcifying invertebrates, including coral, mollusk, crustacean, and echinoderm animals [1,2]. OA impairs not only the ability of calcifying animals to build and maintain their shells and skeletons, but also the physiological processes such as respiration, metabolism, and reproduction in both calcifying and non-calcifying marine invertebrates [3,4]. Illumina short-read RNA-seq (srRNA-seq) has been applied to investigate the transcriptional changes in marine invertebrates under acidic pH environments [5,6]. For instance, acidic pH halted the shell formation in the clam *Ruditapes philippinarum* larvae by simultaneously downregulating Ca^2+^ transporters and carbonic anhydrase genes, depleting triacylglycerol energy reserves, and remodeling membrane lipids [6]. Although various molecular responses, including antioxidation and detoxification enhancement, metabolism remodeling, immune response activation, and calcification in response to OA, were discovered, the post-transcriptional regulatory mechanisms underlying acid stress response in marine invertebrates remain elusive [7].

However, investigating the post-transcriptional activities of genes has been hampered by short-read RNA sequencing (srRNA-seq) [8]. Strikingly, long-read RNA sequencing (lrRNA-seq) has demonstrated the ability to resolve the full complexity of mRNA isoforms and integrate multiple post-transcriptional events with transcriptional networks [9]. The emerging lrRNA-seq technologies based on PacBio and Oxford Nanopore (ONT) platforms are well-suited to identify novel splice variants, non-canonical junctions, and fusion transcripts, as well as novel regulatory networks [10,11,12]. Alternative splicing of innate immunity-related genes identified by PacBio lrRNA-seq was identified in Pacific abalone, *Haliotis discus hannai*, in response to adverse environments [13]. In addition, lncRNAs of Scleractinia coral *Pocillopora damicornis*, detected by ONT lrRNA-seq, were found to promote phenotypic plasticity for enhancing heat tolerance [14]. Furthermore, the response to different salinity conditions in the marine nematode *Litoditis marina* was investigated using ONT lrRNA-seq, and its isoform-level alterations, including transcript usage and alternative polyadenylation (APA), were reported [10].

Our previous studies based on Illumina srRNA-seq revealed the divergent acidic pH stress responses of acid-tolerant terrestrial nematode *Caenorhabditis elegans* and more acid-sensitive marine nematode *L. marina* [15,16]. Specifically, *C. elegans* can tolerate severe acidity (pH ≤ 2.93), while *L. marina* failed to develop into adults under pH 4.33 or survive under pH 3.33. Fatty acid metabolism and P450 detoxification pathways contribute to acid pH response of both *C. elegans* and *L. marina*, yet the post-transcriptional regulation remains to be elucidated.

This bacterivorous free-living *L. marina* (formerly *Pellioditis marina* or *Rhabditis marina*) [17] is particularly notable due to its short life cycle and access to comprehensive genome sequences, annotations and functional genomics tools, which enable *L. marina* to be readily maintained and propagated under controlled laboratory settings, making it an ideal marine model organism for the comparative study of the impacts of acidic pH stress on non-calcifying marine invertebrates [18], as well as responses to anthropogenic pollutants [19,20]. *L. marina* represents a cryptic species complex within the family Rhabditidae that has emerged as a powerful model for investigating fundamental questions in nematode evolution, phylogeography, and community ecology [21]. Inhabiting decaying macroalgae in intertidal zones [22], this morphospecies comprises at least ten genetically distinct cryptic species (Pm I–Pm X) that frequently co-occur sympatrically [22,23,24], challenging traditional competitive exclusion principles through mechanisms of niche partitioning, microhabitat differentiation, and differential responses to environmental stressors such as temperature and salinity [23,24,25,26]. These cryptic lineages diverged millions of years ago [27], yet exhibit physiological and behavioral divergence, including specific dispersal strategies [28,29] and temperature-related fitness [30].

In addition, marine nematodes can also be a promising live feed for an aquacultural hatchery. Nematodes such as *Panagrolaimus* sp. have been successfully applied as live feeds in aquaculture [31], suggesting *L. marina* can potentially be used as a natural alternative to Artemia for shrimp, marine fish larvae, and bivalve spat [32], which can lower artificial breeding costs and provide a stable batch supply for aquaculture hatcheries.

Leveraging ONT lrRNA-seq, we conducted analyses including differential gene expression (DEG), differential transcript expression (DET), differential transcript usage (DTU), differential alternative splicing (DAS), and APA in *L. marina* to investigate the antioxidant strategies and post-transcriptional regulation under acidic pH stresses, which can inform stress mitigation and quality control protocols in aquaculture species, as well as developing acidification-resistant broodstocks.

## 2. Materials and Methods

### 2.1. Sample Maintenance, Collection, and Library Preparation

The marine nematode *L. marina* used in this study was Pm III, which was isolated from the intertidal sediments of Huiquan Bay, Qingdao (36°03′ N, 120°21′ E) in 2016 [33]. Since the isolation, *L. marina* had been cultured on seawater nematode growth medium (SW-NGM) plates seeded with *E. coli* OP50 at 20 °C in our laboratory. Prior to the collection processes, *L. marina* was cultured on SW-NGM plates seeded with 15 × *E. coli* OP50 at 20 °C [10].

For the collection process, adults were removed by rinsing with sterile seawater once they laid abundant eggs. Eggs were then collected by centrifuge, treated with Worm Bleaching Solution, and incubated overnight in sterile seawater for synchronization. The L1 larvae were transferred to fresh SW-NGM plates seeded with 1000 μL of OP50 under three pH conditions (pH 4.33, 5.33, and 5.92 as a control; 3 replicates per condition). After 3 h of growth, worms were immediately collected by rinsing with M9 solution, flash-frozen in liquid nitrogen, and stored at −80 °C. Total RNA was extracted using TRIzol (Invitrogen; Carlsbad, CA, USA). Nine libraries (three biological replicates per treatment) were prepared with the Ligation Sequencing Kit 1D (SQK-LSK109, Oxford Nanopore; Oxford, UK) following the manufacturer’s protocol and sequenced on ONT PromethION using R9 flow (FLO-PRO002).

### 2.2. Isoform Identification and Quantification

The raw reads were processed with Porechop (v0.2.4), and reads with a mean Phred < 7 were discarded. Processed reads were mapped to the genome with IsoQuant (v3.4.1) [11]. Next, mapped reads were collapsed with IsoTools (v0.3.4), and only the “strict” transcripts were retained [34]. Each “strict” transcript was classified relative to WormBase WS270 with IsoTools’ built-in classify module. The isoforms were classified as FSM (full-splice match, perfect match to an annotated intron chain), ISM (incomplete-splice match, subset of an annotated intron chain), NIC (novel-in-catalog, new chain using known splice sites), NNC (novel-not-in-catalog, at least one new splice site), genic, antisense, intergenic, fusion as appropriate.

ONT fastq files were aligned to the newly generated transcriptome annotation with minimap2 (v2.24) [35]. Transcript-level abundance was estimated with Oarfish (v0.3.0) [36]. Transcripts shorter than 200 nucleotides or with zero counts across all libraries were discarded.

### 2.3. Differential Analysis

Differential gene expression analysis was performed with DESeq2 (v1.38) in R 4.3.0 [37]. Transcript-level counts were first summed to gene-level counts by summing all transcript counts that mapped to the same gene. The DESeq2 analysis was initialized with the count matrix and performed with default parameters (betaPrior = FALSE). Contrasts were extracted for each acidic pH condition versus the reference pH condition. Genes with an adjusted *p*-value (Benjamini–Hochberg) < 0.05 and |fold change| > 1.5 were considered differentially expressed.

Differential transcript expression analysis was conducted with DESeq2 (v1.38) with default parameters (betaPrior = FALSE). Transcripts were considered differentially expressed if the Benjamini–Hochberg adjusted *p*-value ≤ 0.05 and |fold change| > 1.5.

Differential alternative splicing analysis was performed with SUPPA2 (v2.3) [38]. First, the transcript-level TPM tables and annotation file with newly assembled transcripts generated above were used to build an event definition file. Events were extracted with “generateEvents” (-e SE SS MX RI FL-f ioe). PSI (percent-spliced-in) values per event and per replicate were then calculated using ‘psiPerEvent’ with the default parameter. Differential PSI (ΔPSI) was estimated with the empirical significance test implemented in diffSplice (1000 permutations, –gc). Events with |ΔPSI| ≥ 0.1 and empirical *p* < 0.05 were considered significant.

Differential transcript usage analysis was performed with SUPPA (v2.3). Local events were extracted with the “generateEvents” module operating in “ioi” (isoform-over-isoform) mode. Transcript-level abundance matrices (TPM) obtained above were then supplied to the “psiPerIsoform” together with the “ioi” file. Next, differential usage between the two biological conditions was tested with the “diffSplice” module. Events were called significant if the empirical *p* < 0.05 and |ΔPSI| ≥ 0.1.

We modified the LATER pipeline for differential 3′ UTR usage analysis [39]. Per-sample counts were aggregated by shared promoter, transcription termination sites (TESs), or promoter–TES pairs. Genes with multiple promoters or terminators were classified as alternative TSS usage or alternative polyadenylation, respectively. TESs were further annotated as proximal, other, or distal. Bin-level counts were analyzed with SUPPA2 to calculate relative 3′-end usage. Events were significant if empirical *p* < 0.05 and |ΔPSI| ≥ 0.1.

### 2.4. Enrichment Analysis

All KEGG, GO, Reactome, and PANTHER enrichment analyses were performed with KOBAS v3.0 using the hypergeometric test (Fisher’s exact test) and Benjamini and Hochberg for FDR correction [40]. WikiPathways enrichment results were obtained from the enrichment module of the STRING database [41]. *C. elegans*-specific gene expression enrichment was performed on the WormExp2 website [42]. The annotation of genes with broad categories was obtained with WormCat 2.0 [43].

We curated a list of 109 genes contributing to epigenetic regulation, including the methyltransferase, demethylase, chromatin remodeling, and other regulatory genes in *C. elegans*, which were used for filtering the mutant datasets from WormExp2 results (Table S2).

The motif enrichment of RI and SE events was performed with XSTREME v1.0 (MEME Suite 5.5.2) with default parameters. Foreground sequences were extracted from the event region of DAS genes and 50 nucleotides upstream and downstream of this region based on the SUPPA2 results. Background sequences that had the same number of sequences as the foreground were random sequence subsampled from the genomes with the same length distribution.

### 2.5. WGCNA

We constructed signed co-expression networks using the WGCNA package in R (version 1.72-1) [44]. Prior to network construction, genes or transcripts with excessive missing values or low variance across samples were filtered out. The remaining expression matrix or usage matrix was used as input for network construction. Signed network was constructed using the blockwiseModules function with the following key parameters: networkType = “signed”, corType = “bicor”, mergeCutHeight = 0.3. Module-trait relationships were assessed by correlating module eigengenes with pH conditions using Pearson correlation. The gray module was excluded from downstream analyses as it represents genes not assigned to any module.

The enrichment of genes in each module was conducted on the KOBAS or String database.

## 3. Results

### 3.1. Differential Expression Responses to Acidic pH Stress in L. marina

**Figure 1:**
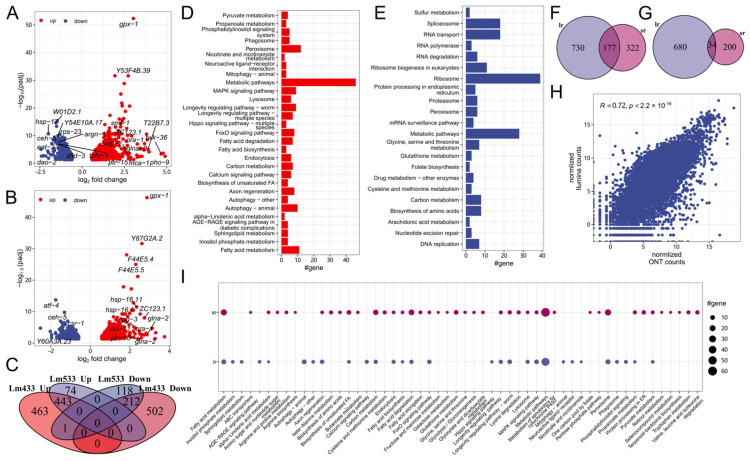
Gene expression response to acidic pH stress in *L. marina*. (**A**) Volcano plot of the significant DEGs of Lm433. (**B**) Volcano plot of the significant DEGs of Lm533. (**C**) Overlaps of DEGs in Lm433 and Lm533. (**D**) KEGG enrichment of the upregulated DEGs in Lm433. The number sign # represents the number. FA, fatty acid. (**E**) KEGG enrichment of the downregulated DEGs in Lm433. (**F**) Overlaps of DEGs from sr- and lr-RNA-seq in Lm433. (**G**) Overlaps of DEGs from sr- and lr-RNA-seq in Lm533. (**H**) Expression of genes under pH 4.33 based on sr- and lr-RNA-seq. Pearson’s *r* and *p*-value were shown. (**I**) Comparison of KEGG enrichment of the upregulated DEGs from sr- and lr-RNA-seq in Lm433.

The ONT lrRNA-seq in *L. marina* was performed under the same acidic pH stress environments as our previous srRNA-seq (pH 4.33 and pH 5.33 as stress conditions, and pH 5.92 as the control condition) [15,16]. We identified 912 significant upregulated and 728 significant downregulated genes under pH 4.33 compared to pH 5.92 (hereafter Lm433), while 521 and 343 significant upregulated and downregulated genes were found under pH 5.33 in comparison to pH 5.92 (hereafter Lm533), respectively (Figure 1A–C, Table S1). Notably, *gpx-1* was among the most significantly upregulated genes under both acidic pH conditions. Under pH 4.33, 29 KEGG pathways, including Fatty acid metabolism, Autophagy, and Peroxisome were enriched in the upregulated DEGs (Figure 1D), of which pathways such as alpha-Linolenic acid metabolism, Glutathione metabolism, and Fatty acid metabolism were related to oxidative stress; while 22 pathways such as Ribosome, RNA transport, Spliceosome and other pathways were enriched in the downregulated genes (Figure 1E). In the alpha-Linolenic acid metabolism pathway, *acox-1.1* was significantly upregulated under both pH 4.33 and 5.33, which might produce more ROS from fatty acid β-oxidation [45]. The downregulated *gpx-5* was related to Glutathione metabolism and Arachidonic acid metabolism pathways, which might increase lipid oxidation [46]. In the Carbon metabolism pathway enriched in the upregulated genes, we identified *sdha-1*, which has been proven to be associated with the production of ROS in different species [47,48]. In the Glutathione metabolism pathway, genes such as *gpx-1*, *gstk-1*, *txdc-12.1* were upregulated, which might promote the resistance to ROS or lipid peroxidation [49,50].

Comparisons between our published srRNA-seq results [15] and lrRNA-seq in this study under pH 4.33 vs. pH 5.92 revealed that, of the 499 upregulated and 234 downregulated DEGs identified by srRNA-seq, 177 and 34 genes were also up- or downregulated in lrRNA-seq, respectively (Figure 1F,G, Table S1) [15]. The expression of genes obtained from the two sequencing platforms under pH 4.33 was highly positively correlated (ρ = 0.72; Figure 1H). Moreover, 17 shared KEGG pathways were identified between the enrichment results of the upregulated DEGs from lrRNA-seq and srRNA-seq, contrasting to one shared KEGG pathway from the downregulated DEGs (Figure 1I, Table S1). In the upregulated DEGs, 12 unique KEGG pathways, including Mitophagy, Autophagy, Phagosome, and Calcium signaling pathway, were enriched in the lrRNA-seq, compared to 25 uniquely enriched pathways, including Glutathione metabolism, Cysteine and methionine metabolism, Lysine degradation, beta-Alanine metabolism, Protein processing in endoplasmic reticulum, and Tryptophan metabolism of srRNA-seq (Table S1). In the downregulated DEGs, 20 pathways, such as Ribosome biogenesis in eukaryotes, Ribosome, RNA transport, and Spliceosome, were only enriched in lrRNA-seq, while only 2 pathways (Phenylalanine metabolism; Phenylalanine, tyrosine and tryptophan biosynthesis) were uniquely enriched in srRNA-seq (Table S1).

Next, we focused on the GO enrichment results. From lrRNA-seq, we found that terms such as response to wounding, phosphatidylinositol phospholipase C activity, carnitine metabolic process, phagocytic vesicle membrane, activation of MAPKK activity, negative regulation of lipid storage and others (*n* = 326) were enriched in the upregulated genes of Lm433, while terms including U5 snRNP, MCM complex, DNA strand elongation involved in DNA replication, ribosomal large subunit biogenesis, formation of cytoplasmic translation initiation complex, eukaryotic 48S preinitiation complex, box C/D snoRNP complex, snoRNA binding and others were enriched in the downregulated genes of Lm433 (*n* = 263; Table S1). In addition, we identified 94 shared GO terms between the enrichment results of the upregulated DEGs from lrRNA-seq and srRNA-seq in Lm433, contrasting to 11 shared GO terms of the downregulated DEGs (Table S1). Together, our data indicated that lrRNA-seq could complement srRNA-seq at the gene expression level.

### 3.2. WormExp2 Analysis Reveals Genes Which Might Regulate Acidic Stress

**Figure 2:**
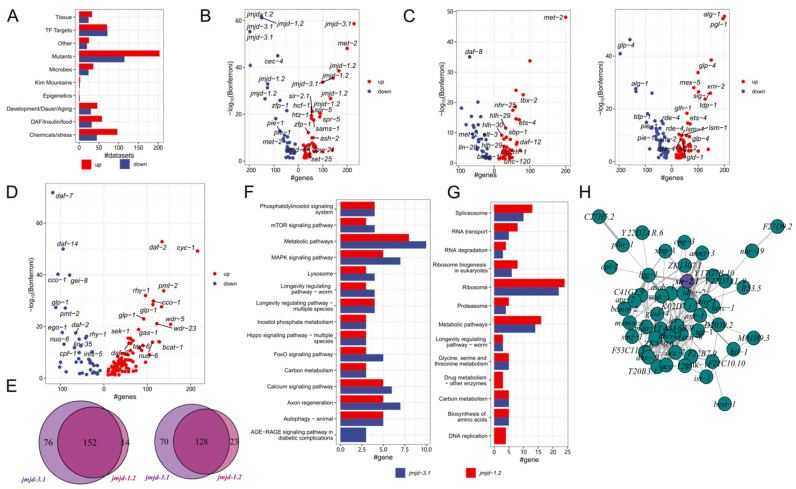
WormExp2 enrichment of DEGs. (**A**) The number of datasets in different categories enriched in the DEGs of Lm433. (**B**) Top enriched Mutants datasets related to epigenetic regulation in Lm433; (**C**) top enriched Mutants datasets related to TFs (**left**) and RBPs (**right**) in Lm433. (**D**) Top enriched Mutants datasets not related to regulators in Lm433. (**E**) Overlaps of the target genes of *jmjd-3.1* and *jmjd-1.2* from the upregulated (**left**) and downregulated (**right**) genes in Lm433. (**F**) KEGG enrichment of the target genes of *jmjd-3.1* and *jmjd-1.2* from the upregulated genes in Lm433. (**G**) KEGG enrichment of the target genes of *jmjd-3.1* and *jmjd-1.2* from the downregulated genes in Lm433. (**H**) genes targeted by *sir-2.1* (purple) in the upregulated genes (green) of Lm433.

We performed WormExp2 enrichment on the DEGs to identify mutants exhibiting similar transcriptional changes with our lrRNA-seq analysis [42]. In Lm433, 574, and 366, the datasets were significantly enriched in the upregulated and downregulated DEGs, respectively (Figure 2A, Table S2). Among these datasets, 204 mutants, 71 TF targets, and 97 Chemicals/stress datasets were enriched in the upregulated DEGs, while 115 mutants, 72 TF targets, and 45 Chemicals/stress datasets were enriched in the downregulated DEGs. Among 204 mutants enriched in the upregulated genes, we identified 45 mutants related to epigenetic regulation (see Section 2.4), 44 transcription factor (TF) mutants, 61 RNA-binding proteins (RBPs) mutants, and 7 splicing factors (SFs) mutants (Table S2). In contrast, we identified 37 mutants of epigenetic regulators, 30 mutants of TFs, 50 mutants of RBPs, and 3 mutants of SFs from the 115 datasets enriched in the downregulated genes (Table S2).

The most significantly enriched datasets in the upregulated DEGs related to epigenetic regulators involved genes such as *jmjd-3.1*, *met-2*, *jmjd-1.2*, *sir-2.1*, and *spr-5*, while *jmjd-3.1*, *cec-4*, *jmjd-1.2*, *zfp-1*, and *pie-1* were enriched in the downregulated genes (Figure 2B, Table S2). In comparison, the most significantly enriched datasets of TFs in the upregulated DEGs were related to *met-2*, *tbx-2*, *nhr-25*, *ets-4*, and *hlh-30*, whereas *daf-8*, *elt-3*, *met-2,* and *lin-28* were enriched in the downregulated genes (Figure 2C, Table S2). The most significantly enriched RBP datasets were in the upregulated genes related to *alg-1*, *pgl-1*, *glp-4*, and *mex-5*, while RBPs such as *glp-4*, *alg-1*, *rdp-1*, *pie-1*, and *rde-4* overlapped with downregulated genes (Figure 2C, Table S2).

Among the remaining 94 mutant datasets enriched in the upregulated genes, the most significantly enriched datasets were *daf-2*, *cyc-1*, *pmt-2*, *rhy-1*, *cco-1*, and *glp-1* (Figure 2D, Table S2). In contrast, *daf-7*, *daf-14*, *cco-1*, *gei-8*, *glp-1*, and *pmt-2* were identified in the remaining 40 mutant datasets enriched in the downregulated genes (Figure 2D, Table S2).

Among these datasets related to epigenetic regulators, datasets related to *jmjd-3.1* and *jmjd-1.2* expression or overexpression had the most DEGs, and most of the DEGs in these datasets were identical (Figure 2E, Table S2). Specifically, the DEGs UP by *jmjd-3.1* expressed overlapped with 228 upregulated DEGs of Lm433, and the DEGs from UP by *jmjd-1.2* neuron expressed overlapped with 166 upregulated DEGs of Lm433. A total of 152 genes in the 2 overlaps were identical. The downregulated DEGs of Lm433 overlapped with 151 DEGs from down by *jmjd-1.2* expressed and 198 from down by *jmjd-3.1* expressed, with 128 genes being identical in the 2 overlaps. These upregulated DEGs related to *jmjd-3.1* and *jmjd-1.2* were involved in the MAPK signaling pathway, Axon regeneration, Calcium signaling pathway, FoxO signaling pathway, and Autophagy, while the downregulated DEGs were related to Ribosome, Spliceosome, Ribosome biogenesis in eukaryotes, and RNA transport (Table S2). Notably, DEGs from UP by *sir-2.1*(O/E) overlapped with 57 upregulated DEGs in Lm433, which formed an interacting network containing genes related to autophagy (e.g., *atg-2* and *lgg-1*), mitochondrion (e.g., *icl-1*), fatty acid metabolism (e.g., *acs-7*), and other pathways (Figure 2H, Table S2).

In Lm533, 426, and 194 datasets were significantly enriched in the upregulated and downregulated DEGs, respectively (Table S2). Mutant datasets of *cyc-1*, *alg-1*, *daf-2*, *cco-1*, *pgl-1*, *mex-5*, *glp-1*, *glp-4*, *sig-7*, *hlh-29*, *tdp-1*, *pmt-2*, *rhy-1*, *wdr-5*, *nhr-62*, *eat-2* were the most significantly enriched datasets in the upregulated DEGs, while *daf-7*, *gei-8*, *glp-4*, *cco-1*, *rde-4*, *pmk-1*, *mex-3*, *skn-1*, *tdp-1*, *alg-1*, *cde-1*, *glp-1*, *ego-1*, *xrn-2*, *tom-1*, *nhr-62*, *eat-2*, *nduf-6* were the most significantly enriched in the downregulated DEGs (Table S2). In the Mutant dataset genes of upregulated DEGs (e.g., *cyc-1*, *alg-1*, *daf-2*), 22 pathways, including Axon regeneration, Longevity regulating pathway, Oxidative phosphorylation, MAPK signaling pathway, FoxO signaling pathway and mTOR signaling pathway were enriched, while 15 pathways such as Oxidative phosphorylation, Axon regeneration and Longevity regulating pathway were enriched in the Mutant dataset genes of the downregulated genes (Table S2).

Notably, we found that 5 datasets related to UV (down by UV and TiO2 (Choi), down by UV (Choi), down by UV irradiation on *xpa-1* mutant, down by UV irradiation, UP by UV irradiation on *xpa-1* mutant) were enriched from the upregulated genes of Lm433, and 2 datasets (UP by UV irradiation, *daf-2/daf-16* dependent; UP by UV irradiation) were enriched in downregulated genes of Lm433. Since UV can induce ROS, we inferred that the acidic pH might induce expression changes related to ROS [51]. In addition, both up- and downregulated DEGs overlapped with the DEGs from the dataset UP by 0.1 mM paraquat, which can increase antioxidant enzyme activity in *C. elegans* [52]. In addition, the upregulated DEGs of Lm433 overlapped with the DEGs in the down by rotenone after 5 days datasets, suggesting acidic pH stress might induce mitochondrial ROS production [53].

### 3.3. Weighted Co-Expression Network Analysis (WGCNA) Under Acidic pH Stress

**Figure 3:**
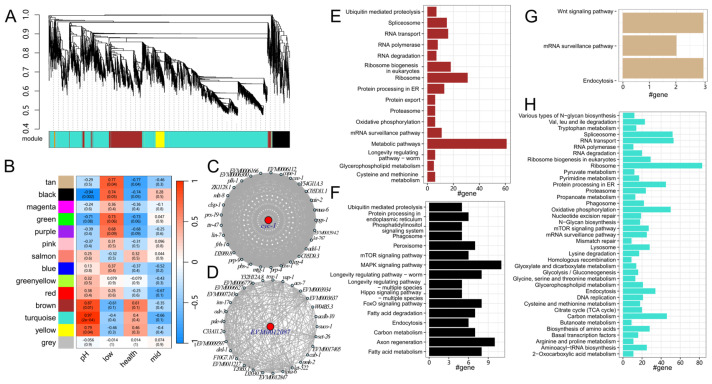
WGCNA on gene expression under acidic pH stress. (**A**) Clusters of genes based on their expression. Each distinct color denotes a unique co-expression module. The *Y*-axis shows unitless TOM-based dissimilarity. (**B**) Correlation between modules, pH, and the health of *L. marina*. pH, a binary indicator (0/1) of whether *L. marina* individuals could develop into adulthood; low, low pH of 4.33; health, the health condition of *L. marina*; mid, pH of 5.33. (**C**) The genes with the highest connectivity in the turquoise module (the hub gene of this module is at the center). (**D**) The genes with the highest connectivity in the black module. (**E**) KEGG enrichment of genes in the brown module. (**F**) KEGG enrichment of genes in the black module. (**G**) KEGG enrichment of genes in the tan module. (**H**) KEGG enrichment of genes in the turquoise module.

We performed WGCNA for analyzing gene expression [44]. A total of 13 modules were identified in this network, in which the turquoise, blue, and brown modules had over 1000 members, while the salmon module had only 94 genes (Figure 3A, Table S3). We inferred the correlation between the alterations and pH gradients, and low pH (pH 4.33), medium pH (pH 5.33), and control conditions (Figure 3B, Table S3). The tan and brown modules were the modules with the highest positive and negative correlation with pH 4.33, respectively. In comparison, the black and turquoise modules were the modules with the highest negative and positive correlation with pH gradients, respectively. In addition, the hub genes of the tan, brown, black, and turquoise modules were *rgl-1*, *F10E9.4*, *EVM0012087*, and *cyc-1*, respectively (Figure 3C,D, Table S3).

Specifically, 26 pathways, including Protein processing in endoplasmic reticulum, Spliceosome, mRNA surveillance pathway, RNA transport, Ribosome biogenesis in eukaryotes, Oxidative phosphorylation, Cysteine and methionine metabolism, Mitophagy, and Apoptosis, were enriched in the brown module (Figure 3E, Table S3). In the black module, 33 pathways such as the MAPK signaling pathway, Axon regeneration, Fatty acid metabolism, FoxO signaling pathway, Longevity regulating pathway, Carbon metabolism, and Peroxisome, were enriched (Figure 3F, Table S3). Only 3 pathways (mRNA surveillance pathway, Endocytosis, Wnt signaling pathway) were enriched in the tan module (Figure 3G, Table S3). The turquoise module, the largest module in this network, contained genes related to 12 pathways, such as Endocytosis, Axon regeneration, MAPK signaling pathway, and Glycerophospholipid metabolism (Figure 3H, Table S3).

In addition, the turquoise module contained 548 (76.75%) and 310 (93.66%) downregulated genes of Lm433 and Lm533, respectively (Table S3). The green module had the most upregulated genes of both Lm433 and Lm533, in which Autophagy, Lysosome, Peroxisome, FoxO signaling pathway, Longevity regulating pathway, Fatty acid metabolism, MAPK signaling pathway, Ubiquitin mediated proteolysis, and Longevity regulating pathway were enriched (Table S3).

### 3.4. Isoform-Level Responses to Acidic pH Stress

ONT lrRNA-seq of *L. marina* under three pH conditions yielded 214,696 collapsed transcripts via IsoTools [34], from which stringent quality control produced 44,317 isoforms across 15,314 genes (10,866 with novel isoforms) (Table S4).

**Figure 4:**
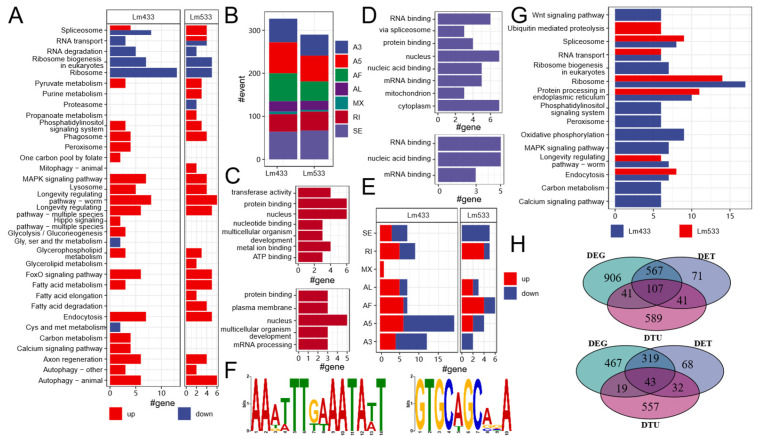
The splicing landscape under acidic pH stress in *L. marina*. (**A**) KEGG enrichment of genes with DETs under acidic pH stress. (**B**) Number of DAS events under acidic pH stress. (**C**) GO enrichment of genes with differential RI events in Lm433 (**top**) and Lm533 (**bottom**). (**D**) GO enrichment of genes with differential SE events in Lm433 (**top**) and Lm533 (**bottom**). Via spliceosome refers to regulation of alternative mRNA splicing, via spliceosome. (**E**) Overlaps between DEGs and genes with DAS events. (**F**) Motifs enriched on the skipped exons (**right**) and retained introns (**left**) in Lm433. (**G**) KEGG enrichment of genes with significant DTU events under acidic pH stress. (**H**) Overlaps among DEG and genes with DET and DTU in Lm433 (**top**) and Lm533 (**bottom**).

ONT lrRNA-seq identified 986 and 537 DETs, 327 and 290 DAS events, as well as 1512 and 1241 instances of DTU events in Lm433 and Lm533, respectively (Table S4). Longevity regulating pathway, MAPK signaling, and Autophagy were enriched in upregulated DETs of Lm433, while Ribosome and Spliceosome in the downregulated DETs (Figure 4A, Table S4). Among the DAS events, exon skipping (SE) and alternative 5′ splice sites (A5) predominated (Figure 4B, Table S4). Genes with intron retention (RI) events related to multicellular organism development, while SE-associated genes enriched in RNA and mRNA binding (Figure 4C,D, Table S4), with 51 and 22 DEGs exhibiting DAS events in Lm433 and Lm533 respectively (Figure 4E, Table S4). Motif analysis of SE and RI events in Lm433 identified an AU-rich motif resembling MDL-1 (UP00382_1) enriched in RI events, and a GA-rich motif similar to MXL-1_MDL-1 (UP00373_2) in SE events (Figure 4F). Additionally, DTU events accounted for 778 genes related to Ribosome, Oxidative phosphorylation, and Spliceosome (Figure 4G, Table S4).

Notably, we observed an extensive overlap between DEG and genes with DETs, indicating that gene-level expression changes were highly correlated with the altered abundance of specific transcripts. In contrast, DAS and DTU exhibited lower overlap with DEG (~30–40% and ~5–10%, respectively; Figure 4H, Table S4), demonstrating that post-transcriptional regulatory mechanisms operated partially independently of transcriptional activation.

### 3.5. APA Contributes to Acidic pH Stress Response

**Figure 5:**
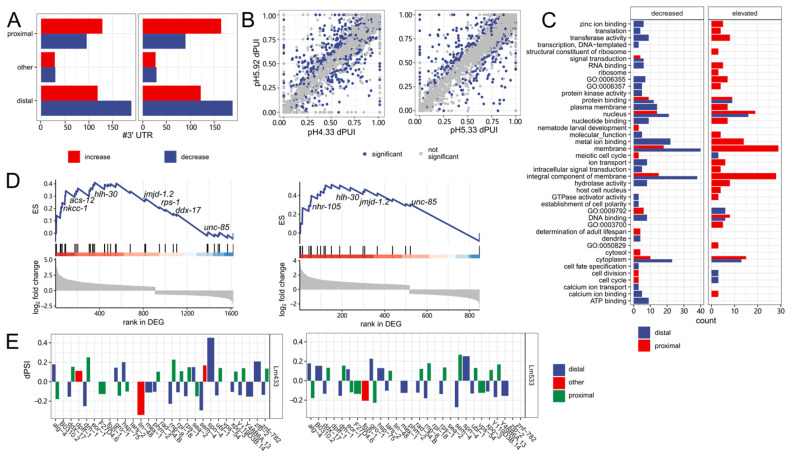
Alteration in 3′ UTR usage under pH stress. (**A**) Numbers of 3′ UTR with differential usage. Distal, a distal 3′ UTR; proximal, a proximal 3′ UTR; other, a 3′ UTR that is in the middle of the distal and proximal 3′ UTRs. (**B**) Differential 3′ UTR usage represented by DPUI. (**C**) GO enrichment of genes with differential 3′ UTR usage. GO:0006355, regulation of DNA-templated transcription; GO:0006357, regulation of transcription by RNA polymerase II; GO:0009792, embryo development ending in birth or egg hatching; GO:0003700, DNA-binding transcription factor activity; GO:0050829, defense response to Gram-negative bacterium. (**D**) DEGs with differential 3′ UTR usage events of Lm433 (**left**) and Lm533 (**right**). (**E**) RBP genes with differential 3′ UTR usage of Lm433 (**left**) and Lm533 (**right**).

APA generates isoforms with identical coding sequences but different 3′ UTR lengths, impacting gene expression and responding to environmental stresses across Metazoa [54,55,56]. We identified 593 significant differential 3′ UTR usage events in Lm433 and 622 events in Lm533 (Table S5). Specifically, 400 genes had significantly differential 3′ UTR usage, including 149 genes preferring distal 3′ UTRs and 214 genes preferring proximal 3′ UTRs in Lm433, contrasting to a total of 414 genes with differential 3′ UTR usage, 151 genes preferring distal 3′ UTRs and 227 genes preferring proximal 3′ UTRs in Lm533 (Figure 5A). We also calculated the distal polyA usage index (DPUI) to represent the preference of the distal 3′ UTR for all the isoforms with multiple polyA sites. A lower average DPUI was identified under pH 4. 33 vs. pH 5.92 (two-sample paired *t* test, *p* < 1.90 × 10^−14^; Figure 5B), which was consistent with the results of our method for investigating the change in 3′ UTR usage on the isoform-level (Figure 5A).

In Lm433, Mucin type O-glycan biosynthesis, Ribosome, and Phagosome were enriched in genes with elevated usage of proximal 3′ UTRs, while Mucin type O-glycan biosynthesis, RNA polymerase, and Phagosome were enriched in genes with decreased usage of distal 3′ UTRs (Table S5). However, no KEGG pathways can be significantly enriched from the genes with elevated usage of distal 3′ UTRs or decreased usage of proximal 3′ UTRs Lm433. GO terms such as DNA binding, protein binding, nucleus, cytoplasm, cell cycle, embryo development ending in birth or egg hatching, cell division and meiotic cell cycle were enriched in genes with elevated usage of distal 3′ UTRs, while signal transduction, nematode larval development, protein binding, nucleus, cytoplasm, cytosol, cell cycle, determination of adult lifespan, embryo development ending in birth or egg hatching, membrane, integral component of membrane, cell division and meiotic cell cycle were enriched in genes with decreased usage of proximal 3′ UTRs under Lm433 (Figure 5C and Table S5).

To ask whether alternative 3′ UTR usage robustly impacts the gene expression level, gene set enrichment analysis was implemented on genes with significant alternative 3′ UTR usage against DEGs [57]. A total of 37 overlapping DEGs were found in Lm433 (26 upregulated and 11 downregulated genes), contrasting to 18 upregulated and 2 downregulated genes in Lm533 (Figure 5D and Table S5). In the 26 upregulated DEGs of Lm433, 13 and 10 genes preferred distal and proximal 3′ UTRs, respectively. In the 11 downregulated DEGs of Lm433, 9 and 3 genes preferred proximal and distal 3′ UTRs, respectively. In Lm533, 6 and 10 of the 18 upregulated genes preferred distal and proximal 3′ UTRs, respectively, while the downregulated ones had a gene preferred distal 3′ UTR and a gene preferred proximal 3′ UTR. In addition, GO terms, including GTPase activity and chloride transmembrane transport, were enriched in the DEGs with differential 3′ UTR usage in Lm433 (Table S5).

We also identified RBP genes with differential 3′ UTR usage under both pH 4.33 and 5.33, and found that more RBP genes preferred proximal 3′ UTRs under pH 4.33 and 5.33 (Figure 5E and Table S5).

Regarding the cleavage and polyadenylation complex [58], only *cfim-2* (upregulated) and *suf-1* (downregulated) were DEGs in Lm433, with no significant changes under pH 5.33.

## 4. Discussion

While srRNA-seq has been widely applied to describe gene expression under stress conditions, our results demonstrated that lrRNA-seq was complementary to srRNA-seq. ONT lrRNA-seq demonstrated superior capacity for isoform discovery, successfully identifying 33,844 novel isoforms on 10,866 coding genes, capturing the isoforms generated by alternative splicing. Although our previous srRNA-seq was able to capture the expression of more genes (16,987 vs. 15,314), more DEGs were captured by lrRNA-seq (Table S1). In addition, we found that 28.80% of the DEGs from srRNA-seq overlapped with 12.90% DEGs from lrRNA-seq (Table S1), suggesting that these two platforms were complementary. Under pH 4.33, 17 shared upregulated KEGG pathways were enriched from the DEGs of the 2 platforms (43.59% of lrRNA-seq and 40.48% of srRNA-seq), while ONT lrRNA-seq uniquely identified pathways including mitophagy, autophagy, and calcium signaling. More downregulated DEGs identified in lrRNA-seq also increased the number of enriched pathways (21 vs. 3 pathways), which included ribosome biogenesis, RNA transport, and the spliceosome.

Previous studies in various marine invertebrates have demonstrated that OA can create an imbalance between the production of ROS and the capacity of the redox system, which might lead to oxidative stress and cellular damage. For example, 28 days of exposure to seawater of pH 7.3 significantly increased the level of ROS and malondialdehyde (MDA) in juvenile horseshoe crab *Tachypleus tridentatus*, and induced the changes in superoxide dismutase (SOD, increased then decreased), catalase (CAT, increased then decreased), and glutathione peroxidase (GPX, decreased then increased) [59]. In the intestine of *Litopenaeus vannamei*, 3 days of exposure under pH 6.9 conditions increased the content of ROS and MDA, decreased the activity of SOD and glutathione S-transferases (GST), yet enhanced the activity of CAT [60]. In blue mussels, *Mytilus edulis*, long-term exposure to low pH increased lipid peroxidation and protein oxidation, as well as the activity of SOD and CAT [61]. In the rotifer *Brachionus koreanus*, intracellular ROS initially increased significantly, then returned to basal levels after prolonged exposure to low pH (pH 7.3 and 7.7), while CAT and SOD activities initially decreased but gradually increased [62]. Transcriptome of pteropod *Limacina helicina* antarctica showed that transcripts for glutathione mitochondrial-like protein and cytochrome p450 pathway were significantly downregulated in low pH (pH 7.71) [63]. In this study, we found that some of the redox genes exhibited expression changes under acidic pH stress in *L. marina* (Table S1). For instance, *gpx-1* and *cyp-13A11* were significantly upregulated under pH 4.33 compared with control conditions. However, the SOD and CAT genes exhibited no significant changes, which might contribute to the vulnerability to acidic pH in *L. marina*. Among genes related to oxidative stress response, the cofactor of *nhr-49* and *mdt-15* were significantly upregulated under pH 4.33, suggesting the activation of phase II detoxification genes [64]. In addition, the upregulation of *mlt-7* was observed under pH 4.33, which might promote the cuticle synthesis via H_2_O_2_—consuming collagen cross-linking [65].According to our previous study, the level of 4-hydroxynonenal (4-HNE) was elevated under both acidic pH stress in *L. marina*, suggesting acidic pH might induce ferroptosis [66,67]. As mentioned above, *gpx-1* was upregulated, while *gpx-5* was downregulated, suggesting the occurrence of ferroptosis. Interestingly, *smf-1* upregulated under both conditions, while *smf-3* only downregulated under pH 4.33, indicating the impairment of iron hemostasis [68]. Additionally, the upregulation of genes related to fatty acid metabolism, such as *fat-2*, might increase the production of PUFA, which might lead to ferroptosis and sensitivity to acidic pH [69]. Given that RNAi of *fat-2* increased resistance to osmotic stress in *C. elegans*, we inferred that suppressing lipid peroxidation might increase the resistance to acidic pH in *L. marina* [67,70].

Among the genes with the highest connectivity in the turquoise module from our WGCNA analysis, we identified *trxr-1*, which was upregulated under both acidic conditions. Studies in *C. elegans* demonstrated that *trxr-1* in the major reducing system regulates lysosomal acidification in the intestine and was upregulated under heat shock [71,72]. Thus, we inferred that genes alleviating oxidative stress might promote acidic pH stress resistance.

Previous studies demonstrated that mitochondrial respiration can generate ROS, especially when respiratory chain complexes I and III are damaged, or the coenzyme Q10 biosynthesis is impaired [73]. Multiple Mutants datasets enriched by WormExp2 in the DEGs were related to respiratory chain complexes genes such as *cyc-1, cco-1, isp-1, nuo-5, nuo-6, gas-1, nduf-6, C34B2.8*, and *D2030.4*, which suggested that acidic pH stress might induce ROS in *L. marina*. The DEGs of UP by *cyc-1* RNAi datasets overlapped with 216 upregulated genes and 102 downregulated genes in Lm433, while the DEGs from down by *cyc-1* RNAi overlapped with 87 downregulated genes, which were among the most significantly enriched datasets [74]. The DEGs of UP by *cyc-1* RNAi datasets overlapped with both upregulated (125) and downregulated (54) DEGs of Lm533, while the DEGs from down by *cyc-1* RNAi had no overlaps. Since *cyc-1* is also the hub gene of the turquoise module, we inferred that genes related to *cyc-1* had important roles in acidic pH stress response. Among these 216 upregulated genes, Beta-oxidation of pristanoyl-CoA and Regulation of ion transport were enriched. In comparison, Mitochondrial unfolded-protein response, Ribosome, and rRNA processing were enriched in the 102 downregulated genes, which also overlapped with genes causing larval arrest (WBP:0000059), growth variant (WBP:0000030), and sterile (WBP:0000688). In contrast, the 87 downregulated genes were related to Metabolism of RNA, Proteasome degradation, and Nitrogen compound metabolic process, which might also impact fertility (WBP:0001384) and development (WBP:0000518). Additionally, UP by *clk-1* mutant was enriched in the upregulated DEGs of Lm433, indicating the expression changes in genes responding to higher levels of ROS under acidic pH stress [75,76].

Among the genes with splicing changes, stress-responsive genes were also identified. Significant DAS events included RI events in osmotic stress regulators *kin-29* (Ser/Thr kinase) and *dos-2* (Notch pathway) in Lm433 [77,78], and a SE event in oxidative stress-responsive *lev-11* (tropomyosin) under both pH 4.33 and 5.33 (Table S4) [79,80]. The apoptosis antagonizing transcription factor *aatf-1* exhibited RI under both conditions (Table S4) [81,82], while a SAM synthase *sams-4*, which regulates mitochondrial unfolded protein response (UPR^mt^) [83], showed RI under both conditions, potentially linking sulfur metabolism disruption (e.g., the downregulation of *ethe-1*, *cysl-2*, *ldh-1*, and *ahcy-1*) to oxidative stress (Tables S1 and S4) [84,85,86]. Under pH 5.33, *hrg-1* exhibited an A5 event, which is associated with V-type H^+^-ATPase and release of free heme under acidification [87]. In addition, we found that DAS genes, including *sod-4*, *prx-2*, *coq-2*, *coq-3*, and *cox-6A*, were related to oxidative stress response.

DTU impacted various processes under acidic pH stress. For example, DTU events occurred on stress regulators, including heat shock responsive *hsf-1* [88,89] and UPR^ER^-regulating x*bp-1* (Table S4) [90]. Additionally, *sod-4*, the extracellular SOD gene, had DTU only under pH 4.33, indicating that oxidative stress might be more intense under pH 4.33 compared to pH 5.33 [91]. Under both pH 4.33 and pH 5.33, significant DTU events were identified in the spermidine synthase gene, *spds-1*, which has been reported to be associated with autophagy and longevity induced by fasting in *C. elegans* [92]. Among the genes related to UPR, a gene related to the oligosaccharyltransferase (OST) protein complex, *stt-3*, had DTU events under both acidic conditions (Table S4), suggesting the interaction between the OST complex and UPR^ER^ might regulate stress response beyond pathogen infection [93]. Notably, ER-stress-related *prx-2* had DTU events under both acidic pH conditions (Table S4) [94]. This peroxisomal gene is related to the NAD^+^-SIRT1-ACOX1-H_2_O_2_-NRF2 signaling axis, which enhances peroxisomal function, fatty acid β-oxidation, and autophagy via α-ketobutyrate metabolism and extends lifespan in *C. elegans* [95]. In addition, two genes related to fatty acid metabolism, *acdh-12* and *acs-14*, exhibited DTU events under pH 4.33, while *acdh-12* was significantly upregulated under both pH 4.33 and pH 5.33 (Table S4), confirming the role of fatty acid metabolism in response to acidic pH stress in marine metazoans [7]. Apart from the DAS genes, we identified genes responding to oxidative stress or related to ferroptosis, including *prx-10*, *ctl-2*, *gst-7*, *trx-1*, *mdt-15*, and *fat-2* among the genes with DTU [69,96].

Among the genes with significant differential 3′ UTR usage, only a small fraction (9.25% in Lm433 and 4.82% in Lm533) of them exhibited expression changes under acidic pH stress. For instance, a gene encoding a component of a calcium-activated chloride channel, *best-1*, preferred the proximal 3′ UTR and upregulated under both conditions (Table S5). Its homolog regulates intracellular pH in the intestinal epithelium of humans, suggesting a conserved role of *best-1* in maintaining intracellular pH [97]. Additionally, *rab-7*, which preferred the distal 3′ UTR and upregulated under both conditions, has been reported to be involved in late endosomal trafficking and autophagosome maturation in *C. elegans* (Table S5) [98,99]. An essential histone chaperone, *unc-85*, preferred the proximal 3′ UTR while downregulated under both pH conditions (Tables S1 and S5) [100,101].Although VHA genes exhibited no expressional changes under pH 4.33, the preference for proximal 3′ UTRs in *vha-2* and *vha-3* under both conditions was observed (Table S5). The regulator of VHA genes, *nhr-31*, preferred the proximal 3′ UTR and upregulated under pH 4.33 (Tables S1 and S5) [102]. Moreover, we identified the differential 3’ UTR usage of genes related to oxidative stress. For instance, *cyp-23A1* preferred the distal 3’ UTR under both conditions, which might prevent excessive ferritin induction [103]. *C07E3.9*/PLA2G1B and *C53D5.5/*GGT1, which protect cells against lipid-peroxidation-induced ferroptosis, had decreased usages of the 3′ UTRs in the middle under pH 4.33 (Table S5) [104,105].

This study might inform aquaculture industry practices of stress mitigation and quality control for live feeds and cultured species. The stress-responsive genes, isoforms, and pathways identified in *L. marina*, particularly those related to antioxidant capacity, ferroptosis resistance, and mitochondrial function, provided insights for developing acidification-resilient live feeds and aquaculture broodstocks. In addition, the upregulation of genes including *gpx-1*, *nhr-49*, and *mdt-15* observed in *L. marina* under pH 4.33 can also serve as sensitive indicators of acid stress in marine invertebrates and benefit the water-quality management in hatcheries.

## 5. Conclusions

In summary, our study highlighted the distinct differences in transcriptional responses to acidic pH stress between short-read and long-read RNA sequencing in *L. marina*, a live-feed organism in nearshore hatchery environments and a potential reference species for aquatic invertebrate studies. While short-read RNA-seq captures broad gene expression patterns at higher sequencing depth, long-read RNA-seq reveals extensive isoform diversity and post-transcriptional regulatory mechanisms that remain hidden in conventional analyses. These findings not only advance the understanding of marine invertebrate responses to ocean acidification but also provide insights into isoform-specific regulatory networks in environmental stress adaptation, as well as offering strategies for stress mitigation and quality control in aquaculture systems under OA. Further research is required to validate the functional significance of specific isoform variants and to explore the broader implications of post-transcriptional regulation for climate resilience in marine ecosystems.

## Data Availability

The original data presented in the study are openly available at CNCB(PRJCA058422).

## Supplementary Materials

The following supporting information can be downloaded at: https://www.mdpi.com/article/10.3390/antiox15070862/s1, Table S1: The DEGs under acidic pH stress in *L. marina*; Table S2: The WormExp2 analysis results of DEGs under acidic pH stress in *L. marina*; Table S3: The WGCNA results under acidic pH stress in *L. marina*; Table S4: The isoform-level changes under acidic pH stress in *L. marina*; Table S5: The changes in 3′ UTR under acidic pH stress in *L. marina*.

## Abbreviations

The following abbreviations are used in this manuscript:

ER	endoplasmic reticulum
UPR	unfolded protein response
polyA	polyadenylation
VHA	V-type H^+^-ATPase
APA	alternative polyadenylation
ROS	Reactive oxygen species
SAM	S-adenosylmethionine
UPR^mt^	mitochondrial unfolded protein response
4-HNE	4-hydroxynonenal
MDA	malondialdehyde
SOD	superoxide dismutase
CAT	catalase
GPX	glutathione peroxidase
GST	glutathione S-transferases
